# Ternion Approach of Surgical Coverage in Single Tooth Recession: A Case Report on a Novel Technique

**DOI:** 10.7759/cureus.71700

**Published:** 2024-10-17

**Authors:** Manju Krishnan, Ranjith Maari, Anitha Balaji, Rudhra K, BalaSubramaniam V, Mouniha Sameera

**Affiliations:** 1 Periodontics, Sree Balaji Dental College and Hospital, Chennai, IND; 2 Periodontics, Sree Balaji Dental College And Hospital, Chennai, IND

**Keywords:** coronally advanced flap, free gingival graft, free rotated de-epithelized papilla, single tooth recession, ternion approach

## Abstract

The goal of mucogingival surgery or periodontal plastic surgery is to preserve gingiva, remove aberrant frenum or muscle attachments, increase vestibular depth, and correct the denuded root surface. Recession coverage is indeed a challenging technique, whereas covering the exposed roots is not only important for aesthetic reasons but also for preventing further issues such as tooth sensitivity, root decay, and loss of supporting bone. A coronally advanced flap (CAF) is one of the most common procedures done for recession coverage because of the concept that the coronal advancement of a flap without tension to cover the exposed root surface or CAF with autogenous grafts results in bulk and improves the thickness of the gingival tissue, increasing the stability and aesthetics of the result. This case report describes the ternion approach of surgical coverage in single tooth recession, with a combination of CAF + free gingival graft + free rotated de-epithelized papilla. This approach, which has the advantage of providing high stability to the flap, also results in bulk, increases the tissue thickness, and enhances aesthetic outcomes.

## Introduction

Mucogingival surgery aims to preserve the gingiva, eliminate aberrant frenum or muscle attachments, increase vestibular depth, perform crown lengthening, and address denuded root surfaces [1]. Among the most common mucogingival abnormalities are gingival recession and a lack of keratinized tissue [2], with the former often being associated with the latter [3]. Gingival recession, defined as the apical migration of the gingiva from the cementoenamel junction [4], can result from various etiological factors, including fenestrations, dehiscence of bone, buccally rotated teeth, prominent roots, improper tooth brushing, and poor oral hygiene [5]. In cases of single-tooth recession, treatment modalities, such as the laterally closed tunnel, rotated flap, modified coronally advanced tunnel, and coronally advanced flap (CAF), are influenced by the extent of recession, type of defect, thickness of keratinized tissue, and vestibular depth. When there is insufficient thickness of the gingival tissue, autogenous grafts, such as connective tissue grafts (CTG), free gingival grafts (FGG), de-epithelialized free gingival grafts (DFGG), and platelet-rich fibrin (PRF), can be combined with these flap techniques to enhance the periodontal phenotype and achieve predictable root coverage [1].

The coronally advanced flap (CAF) is regarded as an effective treatment for localized deep gingival recession with favorable esthetic outcomes [6]. This procedure involves the creation of a tension-free, trapezoidal gingival flap that is mobilized and advanced coronally to cover the recession defect, resulting in excellent root coverage. Miller introduced the use of the free gingival graft (FGG) in root coverage procedures in 1985 [7], building on earlier work by Sullivan and Atkins, which demonstrated favorable results [8]. The primary advantage of the FGG is its ability to effectively increase the width of keratinized tissue.

In 2000, Zucchelli and De Sanctis first described the technique of combining a free gingival graft with a coronally advanced flap in a single-stage procedure, which enhanced the stability, function, and esthetics by promoting better integration of the graft with surrounding tissues. Furthermore, the combination of a coronally positioned flap with a free, rotated papilla autograft has proven effective in covering shallow gingival recessions. This technique preserves the vascular periosteum at the recipient site, allowing the covering flap to promote the re-establishment of circulation within the free rotated de-epithelialized papillary autograft. This enhances the bulk and coverage of the denuded root surface [9].

This case report presents a novel approach that combines a coronally advanced flap with two autogenous grafts (free gingival graft (FGG) + free rotated de-epithelialized papillary autograft (FRDP) to treat a Cairo type 1 recession in the upper canine. This technique leverages the consistent and ample blood supply from the interdental papilla to promote better wound healing, reduce postoperative graft morbidity, and encourage early healing and an increase in keratinized tissue without scarring. Follow-up was conducted at baseline, one week, two weeks, and one month postoperatively.

## Case presentation

**Figure 1:**
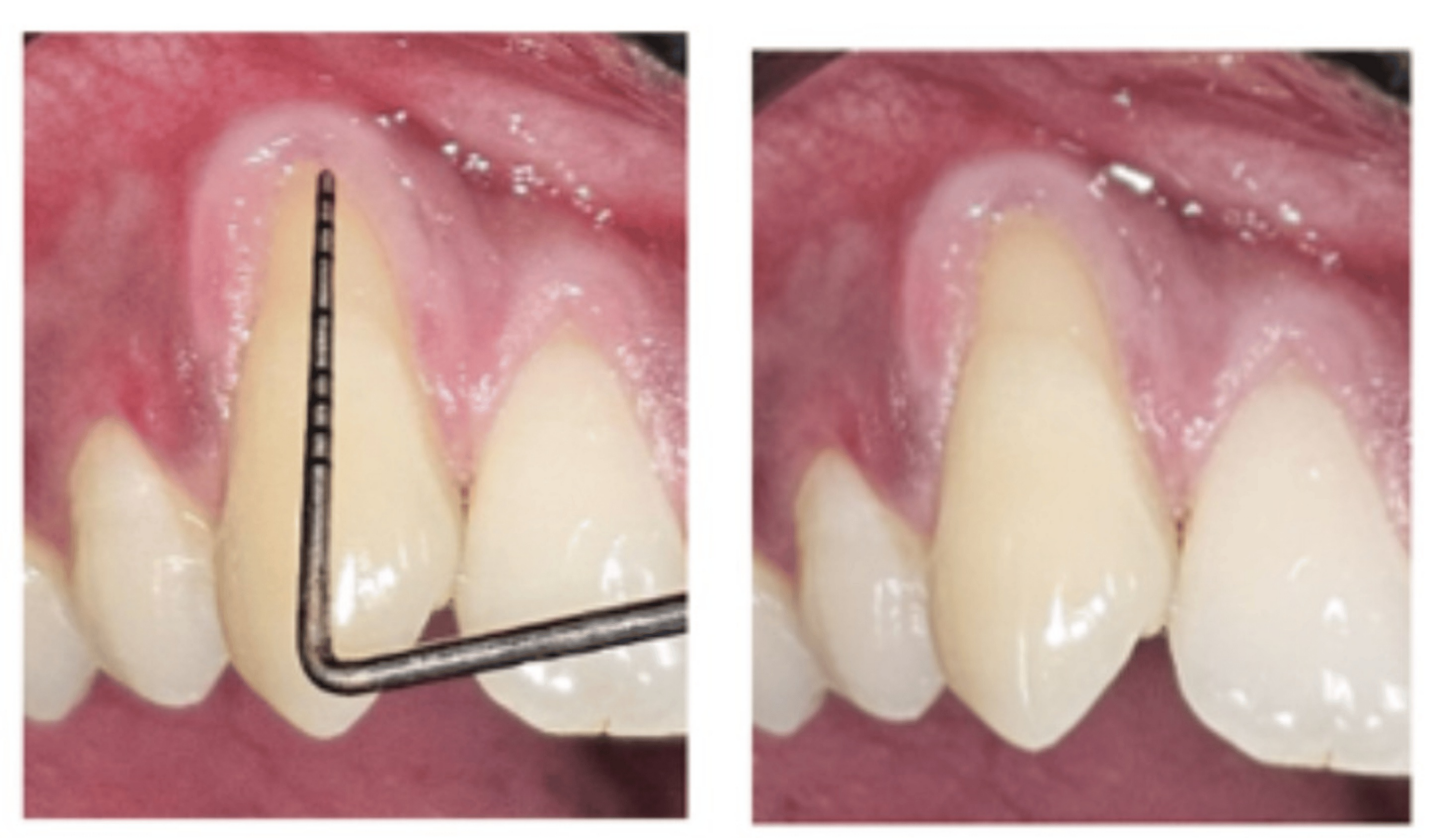
Four mm of clinical attachment loss present

A 32-year-old male patient presented to the Department of Periodontology, Sree Balaji Dental College and Hospital, with the chief complaint of gum recession affecting the upper anterior teeth. The patient reported mild sensitivity in the affected area. His medical history was non-contributory. Upon clinical examination, the patient demonstrated satisfactory oral hygiene, and no periodontal pockets were detected. According to Cairo’s classification, the gingival recession was categorized as RT1 [10], with significant recession observed on the maxillary right canine (Figure 1). Clinical findings included a deep recession with over 4 mm of clinical attachment loss and 1 mm of remaining keratinized tissue at the site of the recession. The primary objective of the treatment was to achieve complete root coverage using mucogingival surgery. The patient did not report any parafunctional habits. Prior to the surgical intervention, oral hygiene instructions, prophylaxis, scaling, and root planing were carried out.

Surgical procedure

**Figure 2:**
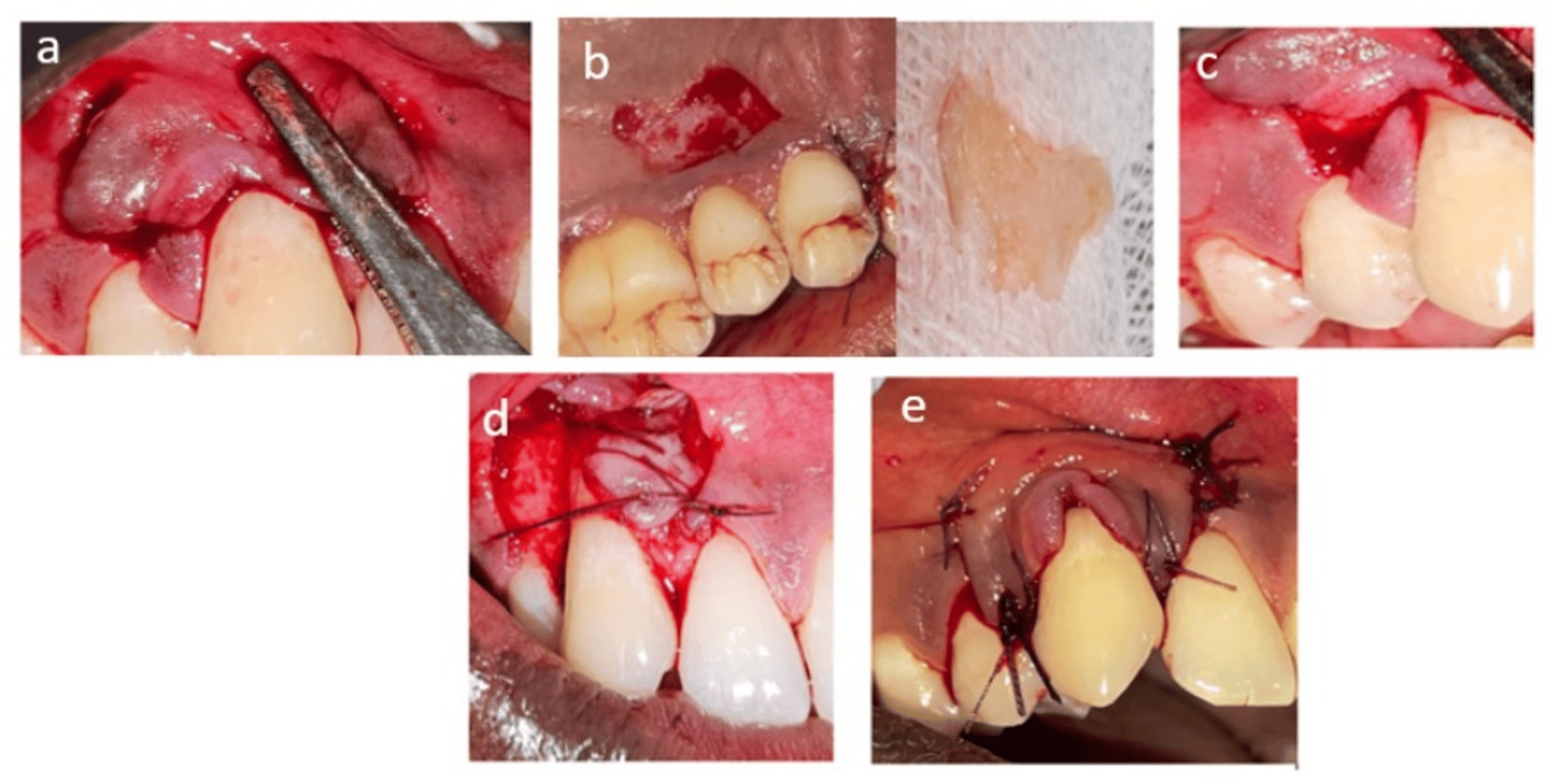
a. Oblique horizontal and vertical incision given; b. Free gingival graft (FGG) harvested from the palate; c. Interdental papillae of the facial aspect will be excised and de-epithelialized; d. The free gingival autograft and interdental papilla autograft were stabilized with a horizontal suture; e. Flap approximated coronally and stabilized with a suture

Local anesthesia was administered using 2% lignocaine hydrochloride with epinephrine (1:200,000). A #15 blade was employed to make precise incisions at the denuded root surface. Two horizontally oblique incisions were made at the level of the cementoenamel junction (CEJ), extending from the line angles of the adjacent teeth on either side of the recession, continuing deep into the papilla. Vertical incisions were made at the distal ends of the horizontal incisions, extending into the alveolar mucosa (Figure 2a).

A partial-thickness flap was elevated by continuously detaching the muscle attachments into the alveolar mucosa, which allowed the flap to be advanced 1 mm coronal to the CEJ to accommodate postoperative tissue shrinkage.

A No. 15C blade was used to harvest a free gingival graft (FGG) from the palatal surface. The graft was collected from the mid-palatal area of the first premolar to the mesial-palatal area of the first molar, maintaining a distance of 1 to 1.5 mm from the gingival margin (Figure 2b). By holding the blade parallel to the tissue, the graft was lifted and separated while maintaining a uniform thickness.

The interdental papilla was detached from its facial aspect, leaving the palatal papilla intact by making V-shaped incisions on the mesial and distal sides of the papilla adjacent to the teeth (Figure 2c). The papilla was de-epithelialized and placed over the defect site in combination with the free gingival graft. Both autogenous grafts were then stabilized with sutures over the denuded root surface (Figure 2d).

Finally, the CAF was positioned 1 mm coronal to the CEJ and sutured inter-proximally using 5-0 vicryl sutures, ensuring no exposure of the grafts (Figure 2e).

**Figure 3:**
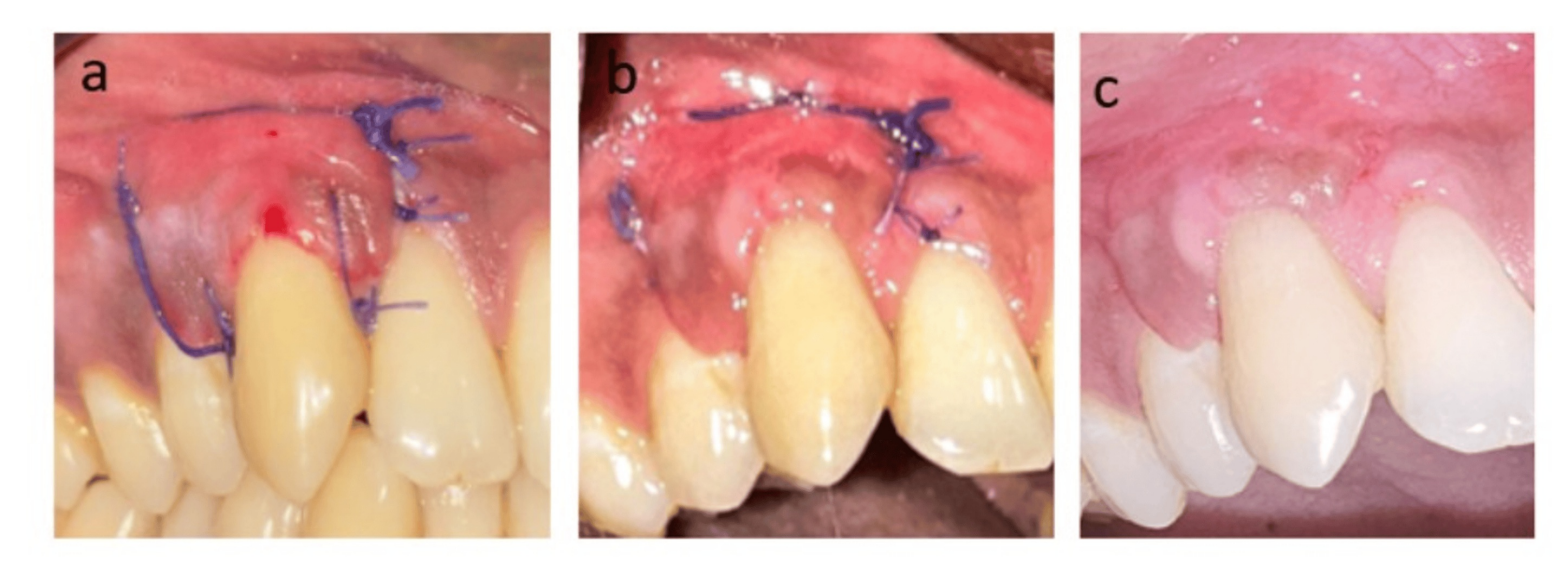
Clinical outcomes during the third day, first week, and second week postoperatively

The clinical outcome was satisfactory during the postoperative period, with consistent improvements observed on the third day (a), first week (b), and second week (c) (Figures 3a-3c).

## Discussion

The surgical management of gingival recession using a combination of a CAF and an FGG is a well-established method for achieving root coverage, increasing tissue thickness, and promoting optimal healing. A critical factor in the success of this procedure is vascularization, which enhances graft survival and improves healing outcomes by supporting growth factors involved in tissue regeneration. Adequate blood supply provides the necessary oxygen and nutrients for the graft to integrate with the recipient site. In this technique, the labial surface of the papilla is de-epithelialized to ensure constant blood flow to the graft. Bruno et al. (2014) emphasize the importance of this step in maintaining continuous vascularization, as insufficient blood flow can lead to graft necrosis and compromised healing [11].

Healing in these procedures is driven by several key growth factors, including vascular endothelial growth factor (VEGF), transforming growth factor-beta (TGF-β), and epidermal growth factor (EGF). VEGF is essential for angiogenesis, which supports revascularization and ensures oxygen delivery to the graft, thereby accelerating integration. TGF-β plays a significant role in collagen maturation, which is crucial for providing structural integrity to the graft site. TGF-β also helps regulate the immune response, reducing inflammation and creating a stable healing environment. EGF also promotes keratinocyte proliferation and differentiation, facilitating re-epithelialization of the graft and contributing to the regeneration of the epithelial layer necessary for long-term stability [12,13].

The combination of FGG with CAF has been widely adopted in treating gingival recession. Zucchelli and De Sanctis (2000) introduced important modifications to the CAF technique, improving incision design and flap positioning, which enhanced the predictability and aesthetic outcomes. The inclusion of the periosteum in the thickness of the flap, as described by Wennström et al., promotes better blood circulation, aiding in graft integration and healing [14].

Historically, the development of root coverage techniques in periodontal surgery has seen significant advancements. The coronally advanced flap was introduced and later refined, improving the procedure's effectiveness. Further refinements enhanced the technique's predictability. Free gingival grafts were employed to extend the vestibular fornix and cover exposed roots. Studies demonstrated the superior outcomes of these grafts in treating gingival recession, emphasizing that grafts placed over recession defects improve vascularization and survival [15,16].

Clinical studies have consistently demonstrated the success of combining CAF with FGG and reported average root coverage of 70% to 90% using this combination technique. Further, Olivier Carcuac et al. (2015) found that incorporating a rotated papilla with CAF resulted in an average coverage of 91.87% for recession defects measuring 2 to 4 mm, highlighting the reliability of this procedure in achieving significant root coverage with natural aesthetic outcomes [17].

## Conclusions

The use of a coronally advanced flap combined with a free gingival graft remains one of the most effective methods for treating gingival recession. The vascularization of the graft, supported by growth factors, such as VEGF, TGF-β, and EGF, plays a critical role in the healing and integration of the graft. The evolution of this technique, with contributions from pioneers, has led to significant improvements in the predictability and success of root coverage procedures.

Clinical outcomes consistently show high levels of root coverage, making this technique a reliable option for both functional and aesthetic management of gingival recession. The combination of CAF with FGGs ensures not only the survival of the graft but also enhances the overall health and appearance of the periodontium.
